# Supplementary material for: Longitudinal Associations of Dietary Sugars and Glycaemic Index with Indices of Glucose Metabolism and Body Fatness during 3-Year Weight Loss Maintenance: A PREVIEW Sub-Study
Source: Nutrients. 2023 Apr 26;15(9):2083. doi: 10.3390/nu15092083 (PMC10180671; doi:10.3390/nu15092083)
Supplement: Supplementary file 1 [file nutrients-15-02083-s001.zip › Suppl_Table S1_DellaCorte.pdf]

Supplementary Table S1

Characteristics of participants by study population at baseline (8 weeks) and 26 weeks (diet and lifestyle outcomes).

	Sydney + Helsinki	Nottingham
N	343	171
**Females (%)**	70.0	56.1
**Age (years)**	58 (50, 64)	55 (43, 63)
**Ethnicity, (%)**		
**Caucasian**	94.5	85.4
**Asian**	2.0	4.7
**African**		9.4
**Arabic**	1.2	0.6
**Hispanic**		
**Other**	2.3	
**Height (m)**	1.68 ± 0.08	1.67 ± 0.09
**BW (kg)**	82.2 (73.4, 94.0)	84.3 (76.0, 96.1)
**BMI (kg/m^2)**	29.0 (26.1, 33.1)	29.8 (27.2, 33.6)
**FM (kg)**	29.9 (22.9, 39.9)	32.9 (25.9, 41.3)
**WC (cm)**	101.6 ± 12.0	101.2 ± 13.0
**Fasting insulin (pmol/L)**	7.5 (5.5, 10.1)	8.5 (5.9, 12.1)
**HbA1c (mmol/mol)**	35.3 ± 3.1	34.8 ± 3.2
**FPG (mmol/L)**	5.8 ± 0.5	5.7 ± 0.6
**C-peptide (pmol/L)**	690 ± 240	729 ± 281
**Energy intake (kcal)**	1670 ± 394	1555 ± 415
**GI**	55.4 ± 7.1	53.1 ± 5.4
**Dietary fiber (g/d)**	25.1 ± 7.8	21.6 ± 7.2
**Protein (E%)**	21.5 ± 4.8	20.8 ± 4.3
**Fat (E%)**	15.0 ± 2.9	13.1 ± 2.8
**Saturated fat (E%)**	5.0 ± 1.5	4.3 ± 1.4
**Carbohydrate (E%)**	39.3 ± 6.9	47.1 ± 8.9
**Total sugars, g/d**	71.6 ± 24.6	77.5 ± 35.8
**Total sugars, en%**	17.5 ± 4.6	20.1 ± 6.7
**Added sugars^, g/d**	17.5 ± 13.5	
**Added sugars, en%**	4.2 ± 2.9	
**Free sugars[#], g/d**		22.5 ± 17.3
**Free sugars, en%**		5.6 ± 3.6

Data are mean ± SD, median (25th, 75th percentiles), or the number of participants (%). *Data were collected at 8 weeks. †Data were collected at 26 weeks. ^Added sugars from Sydney and Helsinki sites. [#]Free sugars from Nottingham site only. *BW, body weight; BMI, body mass index; FM, fat mass; WC, waist circumference; HbA1c, glycated hemoglobin; FPG, fasting plasma glucose; GI, glycemic index*
