# Supplementary material for: Longitudinal Associations of Dietary Sugars and Glycaemic Index with Indices of Glucose Metabolism and Body Fatness during 3-Year Weight Loss Maintenance: A PREVIEW Sub-Study
Source: Nutrients. 2023 Apr 26;15(9):2083. doi: 10.3390/nu15092083 (PMC10180671; doi:10.3390/nu15092083)
Supplement: Supplementary file 1 [file nutrients-15-02083-s001.zip › Suppl_Table S2_DellaCorte.pdf]

Supplementary Table S2 Associations of free sugar, total sugar, and glycemic index with indices of glucose metabolism and body fatness for Nottingham intervention group (*n= 171*)

	**Tertiles of free sugar**				**Tertiles of total sugar**				**Tertiles of glycemic index**			
	**Low (T1)**	**Moderate (T2)**	**High (T3)**	P_{trend}	**Low (T1)**	**Moderate (T2)**	**High (T3)**	P_{trend}	**Low (T1)**	**Moderate (T2)**	**High (T3)**	P_{trend}
Intake (g/d) or GI [a]	8 (5; 11)	19 (16; 21)	39 (36; 42)		44 (42; 46)	70 (68; 72)	108 (106; 110)		48 (47; 48)	55 (54; 55)	61 (60; 61)	
Indices of glucose metabolism												
**Fasting insulin (pmol/L)**												
Model A	9.53 (8.83; 10.23)	9.29 (8.60; 9.98)	9.41 (8.72; 10.11)	**0.032**	10.10 (9.31; 10.89)	9.28 (8.51; 10.05)	9.10 (8.52; 9.69)	**0.024**	9.45 (8.81; 10.09)	8.82 (8.19; 9.46)	10.46 (9.58; 11.33)	**0.005**
Model B	9.77 (9.09; 10.45)	9.23 (8.56; 9.89)	9.25 (8.55; 9.94)	0.364	10.11 (9.32; 10.89)	9.20 (8.45; 9.94)	9.15 (8.57; 9.73)	**0.030**	9.42 (8.80; 10.05)	8.95 (8.34; 9.56)	10.28 (9.42; 11.13)	**0.015**
**HbA1c (mmol/mol)**												
Model A	35.2 (32.3; 38.1)	36.0 (33.1; 38.9)	34.9 (32.0; 37.8)	0.196	35.3 (34.8; 35.8)	36.3 (35.7; 36.8)	34.9 (34.5; 35.3)	**0.038**	35.6 (35.1; 36.0)	35.4 (34.9; 35.8)	35.1 (34.5; 35.7)	0.234
Model B	35.3 (34.9; 35.8)	35.9 (35.5; 36.4)	34.8 (34.3; 35.3)	**0.035**	35.2 (34.6; 36.1)	36.2 (35.3; 37.1)	35.0 (34.6; 36.1)	0.130	35.6 (35.1; 36.0)	35.4 (35.0; 35.9)	35.0 (34.7; 35.6)	0.388
**Fasting glucose (mmol/L)**												
Model A	5.95 (5.62; 6.28)	5.89 (5.57; 6.22)	5.79 (5.47; 6.12)	**0.006**	5.94 (5.86; 6.17)	5.92 (5.82; 6.12)	5.82 (5.72; 6.08)	**<0.001**	5.90 (5.82; 5.98)	5.84 (5.76; 5.92)	5.84 (5.74; 5.94)	0.377
Model B	5.94 (5.85; 6.04)	5.90 (5.81; 5.99)	5.79 (5.70; 5.88)	**0.008**	5.89 (5.84; 6.14)	5.90 (5.75; 6.16)	5.86 (5.71; 6.11)	0.069	5.88 (5.80; 5.96)	5.85 (5.77; 5.92)	5.87 (5.76; 5.97)	0.814
**C-peptide (pmol/L)**												
Model A	751 (714; 789)	728 (691; 765)	712 (675; 749)	0.816	776 (734; 817)	734 (694; 775)	703 (672; 734)	**0.003**	761 (727; 795)	679 (645; 712)	771 (725; 818)	0.186
Model B	761 (725; 798)	724 (689; 759)	706 (669; 743)	0.771	774 (733; 817)	728 (689; 768)	707 (676; 738)	**0.009**	758 (724; 791)	684 (652; 717)	767 (722; 812)	0.079
Indices of Body Fatness												
**Body fat (%)**												
Model A	37.1 (32.3; 41.8)	38.4 (33.7; 43.1)	38.4 (33.7; 43.1)	0.018	38.2 (33.8; 42.6)	38.4 (34.1; 42.8)	37.5 (33.3; 41.8)	0.933	37.9 (36.8; 39.1)	37.4 (36.2; 38.6)	39.0 (37.3; 40.7)	0.105
Model B	37.7 (36.4; 39.0)	38.3 (37.0; 39.6)	37.8 (36.5; 39.2)	0.267	37.6 (36.0; 39.1)	38.3 (36.9; 39.8)	37.9 (36.8; 39.1)	0.190	38.3 (37.1; 39.5)	37.1 (35.9; 38.3)	38.7 (37.0; 40.4)	0.382
**BMI**												
Model A	30.0	29.8	30.0	0.237	30.4	29.7	29.8	0.345	30.1	29.5	30.4	0.225

	(27.7; 32.3)	(27.5; 32.1)	(27.7; 32.4)		(28.3; 32.6)	(27.6; 31.8)	(27.7; 31.8)		(29.5; 30.7)	(28.9; 30.1)	(29.6; 31.3)	
Model B	30.1 (29.4; 30.7)	29.7 (29.1; 30.4)	30.0 (29.3; 30.6)	0.282	30.1 (29.3; 30.8)	29.6 (28.9; 30.3)	30.0 (29.5; 30.6)	0.411	30.0 (29.4; 30.6)	29.4 (28.8; 30.0)	30.6 (29.8; 31.5)	0.085
**Waist circumference (cm)**												
Model A	102.2 (96.1; 108.3)	99.0 (92.9; 105.1)	98.7 (92.6; 104.8)	0.137	102.7 (97.1; 108.3)	99.0 (93.4; 104.6)	99.0 (93.5; 104.4)	**0.004**	100.1 (98.5; 101.7)	98.6 (97.0; 100.2)	102.1 (99.9; 104.3)	0.175
Model B	101.9 (100.2; 103.7)	99.1 (97.4; 100.8)	98.8 (97.0; 100.6)	0.329	102.1 (100.1; 104.0)	98.8 (96.9; 100.7)	99.4 (97.9; 100.9)	0.108	99.5 (98.0; 101.1)	98.8 (97.3; 101.4)	102.8 (100.5; 105.0)	**0.019**

Values are adjusted least-square means (95% CIs) unless otherwise indicated. Linear trends (P_{trend}) were obtained using a linear mixed model with repeated measures. The predictors of glycemic index, dietary free sugar intake and total sugar intake were used as continuous variables. Model A adjusted for age at time of study begin and intervention center. Model B additionally adjusted for body fat percentage (only for glucose metabolism outcomes), energy intake, protein intake, fibre intake and saturated fat intake. All dietary variables were energy-adjusted. Transformations of variables for analysis: $\log_e$ for protein intake, saturated fat intake, energy intake, BMI, HbA1c, insulin and C-peptide; square root for total and free sugar intakes. HbA1c: glycated hemoglobin A1c. BMI: body mass index. [a] Values are unadjusted medians (25th, 75th percentile). *P*-values stem from models with predictors as continuous variables. Bold values indicate significant findings ($p < 0.05$).
