# Supplementary material for: Longitudinal Associations of Dietary Sugars and Glycaemic Index with Indices of Glucose Metabolism and Body Fatness during 3-Year Weight Loss Maintenance: A PREVIEW Sub-Study
Source: Nutrients. 2023 Apr 26;15(9):2083. doi: 10.3390/nu15092083 (PMC10180671; doi:10.3390/nu15092083)
Supplement: Supplementary file 1 [file nutrients-15-02083-s001.zip › Suppl_Table S3_DellaCorte.pdf]

Supplementary Table S3 Associations of fruit intake (g/d) for Helsinki and Nottingham (*n=383*), fruit intake (serv/d) for Sydney (*n=132*), and saturated fat intake for Sydney and Helsinki and Nottingham intervention groups (*n=515*)

	**Tertiles of fruit intake (g/d)**				**Tertiles of fruits intake (serv/d)**				**Tertiles of saturated fat intake (g/d)**			
	**Low (T1)**	**Moderate (T2)**	**High (T3)**	P_{trend}	**Low (T1)**	**Moderate (T2)**	**High (T3)**	P_{trend}	**Low (T1)**	**Moderate (T2)**	**High (T3)**	P_{trend}
Intake (g/d) or GI [a]	59 (51; 68)	179 (170; 187)	353 (344; 362)		0.5 (0.3;0.6)	1.2 (1.0; 1.3)	2.1 (1.9; 2.3)		14 (12; 16)	20 (18; 22)	27 (25; 28)	
**Fasting insulin (pmol/L)**												
Model A	9.55 (9.08; 10.03)	8.96 (8.49; 9.43)	8.91 (8.43; 9.39)	**0.004**	9.33 (8.69; 9.96)	8.98 (8.34; 9.62)	8.34 (7.71; 8.98)	**<0.001**	8.23 (7.69; 8.76)	9.25 (8.71; 9.79)	9.79 (9.24; 10.34)	**<0.001**
Model B	9.34 (8.91; 9.76)	8.89 (8.49; 9.29)	9.17 (8.75; 9.59)	0.284	9.26 (8.18; 9.91)	8.84 (8.10; 9.86)	8.55 (7.49; 9.30)	**0.007**	8.93 (8.14; 9.72)	9.09 (8.43; 9.75)	9.05 (8.26; 9.84)	**<0.001**
**HbA1c (mmol/mol)**												
Model A	36.1 (35.2; 37.0)	35.9 (35.0; 36.8)	35.7 (34.7; 36.6)	**<0.001**	37.0 (36.5; 37.5)	36.9 (36.4; 37.4)	36.3 (35.8; 36.8)	0.071	35.7 (34.8; 36.5)	36.5 (35.7; 37.3)	36.4 (35.6; 37.3)	**<0.001**
Model B	36.0 (34.9; 37.1)	35.9 (34.8; 37.0)	35.7 (34.7; 36.8)	0.073	36.7 (35.1; 38.3)	36.9 (35.4; 38.5)	36.6 (35.1; 38.2)	0.891	35.8 (34.8; 36.7)	36.5 (35.6; 37.4)	36.3 (35.4; 37.2)	0.067
**Fasting glucose (mmol/L)**												
Model A	6.07 (5.86; 6.28)	6.00 (5.77; 6.19)	5.91 (5.70; 6.12)	**0.009**	5.99 (5.90; 6.07)	5.82 (5.73; 5.91)	5.99 (5.91; 6.01)	0.129	5.91 (5.81; 6.03)	5.99 (5.88; 6.10)	6.01 (5.90; 6.12)	**<0.001**
Model B	6.02 (5.80; 6.24)	5.98 (5.76; 6.20)	5.96 (5.74; 6.18)	0.058	5.96 (4.91; 7.01)	5.82 (4.78; 6.87)	6.02 (4.97; 7.06)	0.434	6.00 (5.86; 6.14)	5.97 (5.84; 6.10)	5.93 (5.79; 6.07)	0.382
**C-peptide (pmol/L)**												
Model A	734 (704; 765)	714 (683; 744)	693 (663; 724)	**0.002**	789 (751; 828)	765 (726; 803)	730 (691; 768)	**<0.001**	683 (637; 728)	742 (696; 789)	771 (725; 816)	**<0.001**
Model B	723 (701; 745)	709 (688; 730)	706 (683; 728)	0.118	784 (383; 1185)	756 (355; 1156)	743 (342; 1143)	**0.006**	701 (689; 715)	720 (711; 732)	738 (725; 758)	**<0.001**

Values are adjusted least-square means (95% CIs) unless otherwise indicated. Linear trends (P_{trend}) were obtained using a linear mixed model with repeated measures. The transformed and energy-adjusted predictors of fruit intake (serv/d and grams/d) and saturated fat intake were used as continuous variables. Model A adjusted for age at time of study begin and intervention center. Model B additionally adjusted for body fat percentage, energy intake, protein intake, fibre intake. The fruit intake models additionally adjusted for saturated fat intake and the saturated fat intake model additionally adjusted for glycemic index. Transformations of variables for analysis: $\log_e$ for protein intake, saturated fat intake, energy intake, HbA1c, insulin and C-peptide; square root for total and added sugar intakes. HbA1c: glycated hemoglobin A1c. [a] Values are unadjusted medians (25th, 75th percentile). *P*-values stem from models with predictors as continuous variables. Bold values indicate significant findings ($p < 0.05$).
